# Preparation and Study on Sulfated Nanocellulose/Anthocyanin pH-Sensitive Packaging Materials to Track Food Freshness

**DOI:** 10.3390/foods15030494

**Published:** 2026-02-01

**Authors:** Lan Yang, Qianyu Yuan, Chien-Teng Hsieh, Ching-Wen Lou, Jia-Horng Lin

**Affiliations:** 1 School of Textile Science and Engineering, Tiangong University, Tianjin 300387, China; anyang202210@163.com (L.Y.);; 2 Department of Fashion Design and Merchandising, Shih Chien University, Kaohsiung Campus, Kaohsiung City 845050, Taiwan; 3 Department of Bioinformatics and Medical Engineering, Asia University, Taichung City 413305, Taiwan; 4 Ministry of Education Key Laboratory for Advanced Textile Composite Materials, Tiangong University, Tianjin 300387, China; 5 Advanced Medical Care and Protection Technology Research Center, Department of Fiber and Composite Materials, Feng Chia University, Taichung City 407102, Taiwan

**Keywords:** hydrogel, pH sensor, nanocellulose, anthocyanins, food detection, CNC

## Abstract

Meat products are prone to spoilage during storage and transportation due to the decomposition of amino acids and proteins, which generates volatile amines and elevates pH levels. In recent years, research on pH indicator labels for food has significantly increased. This study investigates a functional film with a bilayer structure for real-time freshness monitoring of meat. Utilizing Tara gum (TG) and Gellan gum (GG) as the base material and nanocellulose composite GG/blueberry anthocyanins as the sensing layer, titanium dioxide was incorporated to effectively block ultraviolet radiation. Through response surface methodology, the cellulose nanocrystals (CNCs) preparation process was optimized to address issues such as insufficient mechanical properties, hydrophobicity, and thermal stability of the composite film. Results showed that the composite film achieved optimal performance when CNC content was 6%. This research provides a simple and effective solution for real-time freshness monitoring of meat products, offering advantages such as low cost, environmental friendliness, and user-friendly operation.

## 1. Introduction

**Figure 1:**
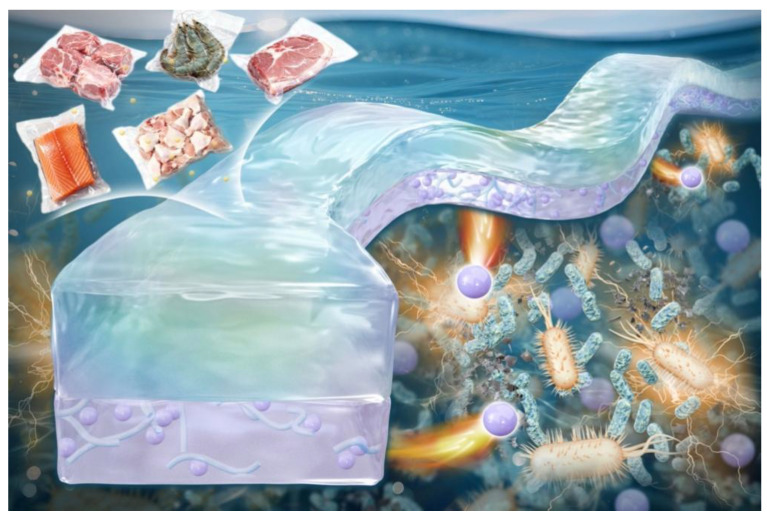
Schematic diagram of pH sensor monitoring for chilled products.

With the continuous improvement of the public’s quality of life and the increasing attention to health, the issue of food safety has increasingly become the focus of the public [1]. Food testing relies heavily on chemical testing for lossy detection, which requires costly specialized tools and experienced personnel [2]. Therefore, the wide application and promotion of pH-responsive intelligent packaging technology provides great convenience and practicability for food quality testing [3,4,5,6]. Smart packaging technology is often used to track the status of food, monitor fluctuations in its quality, and provide consumers with intuitive and easy-to-understand information during the transportation and storage of food [7]. Common colorimetric pH sensors can capture significant color differences, as one of many detection methods, it is extremely consumer-friendly, and its cost is generally more economical. When meat products deteriorate, it may lead to the increase in microorganisms and the production of biogenic amines, such as ammonia, dimethylamine, and trimethylamine, which is also known as total volatile basic nitrogen (TVB-N), so the concentration of TVB-N is an important indicator to assess the freshness of meat [8,9]. Therefore, the use of pH-sensitive display agent to evaluate the content of volatile nitride in meat products has become a feasible method to monitor the freshness of meat [10,11,12]. Figure 1 shows the monitoring schematic of the pH sensor for frozen products. However, a single type of membrane material has various deficiencies in mechanical properties, hydrophobicity, and oxygen permeability [13]. Due to the large specific surface area and surface activity of nanomaterials, they are usually added to compatible polymers to enhance the physical and chemical properties of the materials, such as mechanical strength and thermal stability [14,15]. Because of its high strength, high elastic modulus, good biocompatibility, degradability, and other advantages, nanocellulose is commonly used as a nano enhancer for the packaging film industry, which is used to improve the film material is very sensitive to water, poor mechanical properties, and thermal stability defects.

Therefore, in this study, we first prepared CNC by response surface method to obtain the optimal process conditions, that is, the optimal predicted conditions were as follows: sulfuric acid concentration is 57.25%, temperature is 51.86 °C, and time is 95.65 min. According to the actual conditions of the experiment, the conditions are revised to as follows: sulfuric acid concentration is 57%, temperature is 52 °C, and time is 96 min. Three parallel tests are conducted under optimal conditions, and a good correlation between the predicted value and the experimental value is confirmed. Anthocyanins were extracted from blueberries to develop an anti-ultraviolet double-layer film for monitoring food freshness on the basis of research, after which it was added to the packaging film material as a nano-plasticizer to improve the comprehensive properties of the film material [16]. By measuring the properties of the membrane material, the optimal amount of CNC was determined, and its influence mechanism was analyzed.

## 2. Materials and Methods

### 2.1. Experimental Materials and Instruments

#### 2.1.1. Materials

Blueberries and pork were purchased from the local fresh market (Tianjin, China). Microcrystalline cellulose (MCC) was purchased from Shanghai Yuanye Biotechnology Co., Ltd. (Shanghai, China), low-acyl Gellan gum (GG) was purchased from Shandong Keyuan Biochemical Co., Ltd. (Laizhou, China), and Tara gum (TG) was purchased from Zhejiang Yinuo Biotechnology Co., Ltd. (Hangzhou, China). Rutile titanium dioxide (TiO_2_) nanoparticles (approximately 60 nm in diameter) were purchased from Shanghai Linen Technology Development Co., Ltd. (Shanghai, China). Ethanol, calcium chloride, concentrated sulfuric acid, etc., were purchased from Tianjin Jiangtian Chemical Technology Co., Ltd. (Tianjin, China).

#### 2.1.2. Experimental Instruments

The following experimental instruments were used: electronic balance (ES-E120A, Tianjin Demeng Technology Co., Ltd., Tianjin, China), magnetic stirrer (WH220-HT, Germany WIGGENS, Wuppertal, Germany), CNC ultrasonic cleaner (KQ2200DB, Kunshan Ultrasonic Instrument Co., Ltd., Kunshan, China), Fourier transform infrared spectrometer (FTIR, Nicolet-iS50, Thermo Fisher Scientific, Waltham, MA, USA), contact angle tester (JC2000DM, Shanghai Zhongchen Digital Technology Equipment Co., Ltd., Shanghai, China), scanning electron microscope (SEM, HITACHI-S4800, Japan Hitachi Corporation, Tokyo, Japan), vacuum drying oven (DZF-6020, Shanghai Boxun Industrial Co., Ltd., Shanghai, China), colorimeter (PCE Instruments, Meschede, Germany), vacuum rotary evaporator (Heidolph, Schwabach, Germany), solid–liquid dual-use UV–vis spectrophotometer (UV2700, Japan Shimadzu, Tokyo, Japan), X-ray diffraction instrument (XRD, D8 Discover, Bruker, Karlsruhe, Germany), intelligent electronic tensile testing machine (XLW-PC, Jinan Landlight Electromechanical Technology Co., Ltd., Jinan, China), vacuum freeze-dryer (Scientz-18N, Ningbo Xinzhi Biotechnology Co., Ltd., Ningbo, China), transmission electron microscope (Hitachi H7650, Japan Hitachi Corporation), benchtop high-speed centrifuge (TG16-WS, Hunan Xiangli Scientific Instrument Co., Ltd., Changsha, China), and UV–visible spectrophotometer (VIS-7220N, China, Beijing Ruili Analytical Instrument Co., Ltd., Beijing, China).

### 2.2. Response Surface Analysis for Preparation of Nanocellulose

#### 2.2.1. Preparation of CNC by Acid Hydrolysis

**Figure 2:**
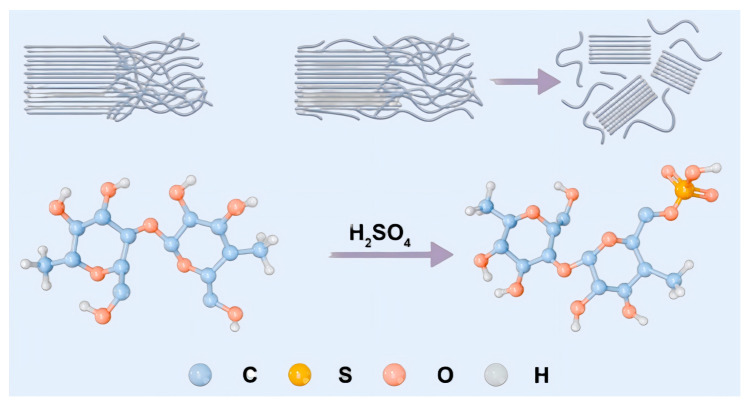
Preparation method of nanocellulose.

The sulfuric acid hydrolysis method utilizes the hydrolytic action of strong acids to remove partial non-crystalline regions in cellulose, followed by ultrasonic treatment to reduce cellulose size. As shown in Figure 2, the process for treating microcrystalline cellulose (MCC) involves mixing 5 g of MCC with concentrated sulfuric acid at varying concentrations at a solid-to-liquid ratio of 8.75 (*m*/*v*) and reacting at a specific temperature. After reaction, the resulting milky white suspension is washed twice with 1 L of distilled water. The gelatinous material collected from the bottom of the centrifuge tube is then placed in a dialysis bag of specific pore size and dialyzed with deionized water until the pH reaches 6–7. Subsequently, the obtained cellulose gel is subjected to ultrasonic disruption (1000 W, 20 min) using an ultrasonic cell disruptor (1000 W). After volume adjustment, the resulting CNC dispersion is pipetted. Twenty milliliters of the volume-adjusted CNC solution are transferred to a pre-weighed volumetric flask and the solution is lyophilized and re-weighed to determine the CNC yield. Three parallel measurements were conducted. The yield calculation is as follows:

w = (m_1_ − m_2_) × V_1_/(m_3_ × V_2_)

In the formula, m_1_ is the total mass of the sample and weighing bottle after drying to a constant weight (g); m_2_ is the mass of the weighing bottle (g); m_3_ is the mass of the raw material (g); V_1_ is the total volume of the CNC after volume adjustment (mL); and V_2_ is the total volume of the CNC transferred to the weighing bottle (mL).

#### 2.2.2. Response Surface Method Design Scheme

The experimental design was conducted according to the Box–Behnken principle. Based on single-factor experiments, the following three critical factors significantly affecting yield were identified: sulfuric acid concentration, temperature, and reaction time. Each factor was set at three levels (−1, 0, and 1). A response surface methodology (RSM) was established with 17 experimental points across this three-factor, three-level system. All experimental data were processed using specialized statistical software to determine the theoretically optimal process parameter combination through model fitting and optimization. Table 1 presents the experimental design for response surface analysis with three factor levels

### 2.3. Preparation of Sulfated Nanocellulose/Anthocyanin pH-Sensitive Packaging Materials

#### 2.3.1. Extraction of Anthocyanin from Blueberry

**Figure 3:**
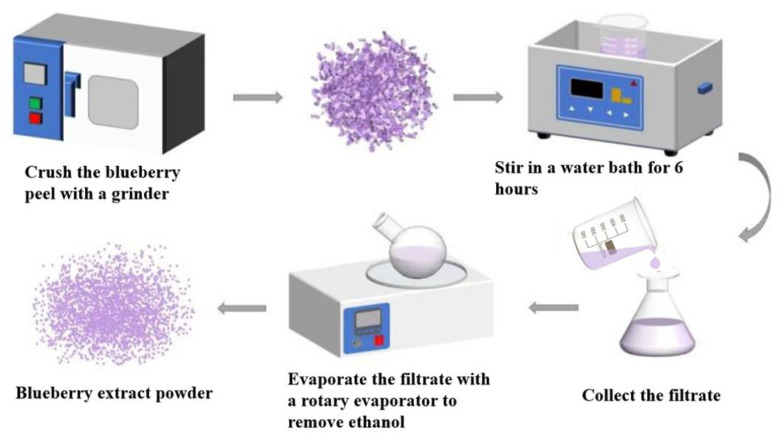
Flowchart of blueberry anthocyanin extraction.

**Figure 4:**
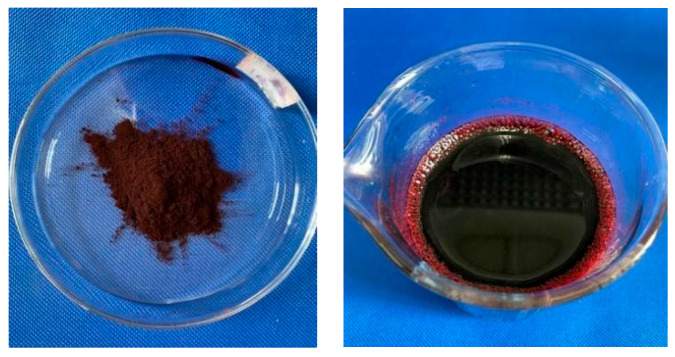
Blueberry anthocyanins.

The dried blueberry skins were crushed and mixed with 80% ethanol solution at a solid-to-liquid ratio of 1:15 (*m*/*v*). The mixture was placed in a constant temperature water bath at 35 °C and continuously stirred for 6 h to extract. After filtration, the filtrate was concentrated under vacuum rotation evaporator (45 °C) in a dark environment to remove ethanol. The concentrated solution was then frozen at −80 °C for 6 h and transferred to a freeze-dryer for continuous drying for 2 days, ultimately obtaining AN extract powder. The powder product was stored in nitrogen-filled brown bottles at 4 °C for subsequent use. The anthocyanin (AN) content was determined by pH differential method [17] and was 612 mg/g. Figure 3 shows the flowchart of blueberry anthocyanin extraction. Figure 4 shows the extracted blueberry anthocyanins.

#### 2.3.2. Preparation of pH Sensor

**Figure 5:**
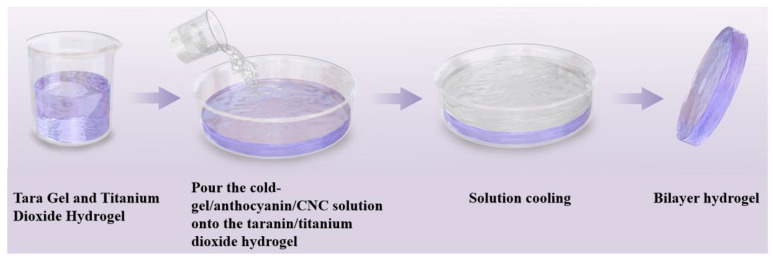
Flowchart of pH sensor preparation.

The double-layer membrane was fabricated using a two-step casting method, as illustrated in Figure 5. First, the lower GG/AN/CNC hydrogel was prepared as follows: 2 g of GG was dissolved in 100 mL water at 98 °C and stirred for 2 h to obtain a clear solution. Under continuous stirring, 5 mL of CaCl_2_ solution (8 mg/mL) was added for ion cross-linking. Subsequently, CNC was added to the solution at concentrations of 0%, 2%, 4%, 6%, and 8% (*w*/*w*, GG) and stirred for 30 min. After cooling the solution to 45 °C, 13 mg of blueberry AN extract powder was added and immediately mixed. The mixture was ultrasonically degassed and poured into a clean 53 mm glass Petri dish, where it cooled at room temperature to form the hydrogel substrate.

Next, prepare the upper TG-TiO_2_ solution and cast it as follows: add 2 g of TG powder to 100 mL of water at 80 °C and stir until dissolved. Ultrasonically disperse TiO_2_ nanoparticles (1.0% mass of TG) in 2 mL of water; then, add the TG solution dropwise. Process the mixture with ultrasonication (10 min) and stirring (10 min). When the mixture cools to 50 °C, immediately pour the TG-TiO_2_ solution onto the prepared GG/AN/CNC hydrogel. After cooling, a rigid yet elastic bilayer hydrogel forms.

**Figure 6:**
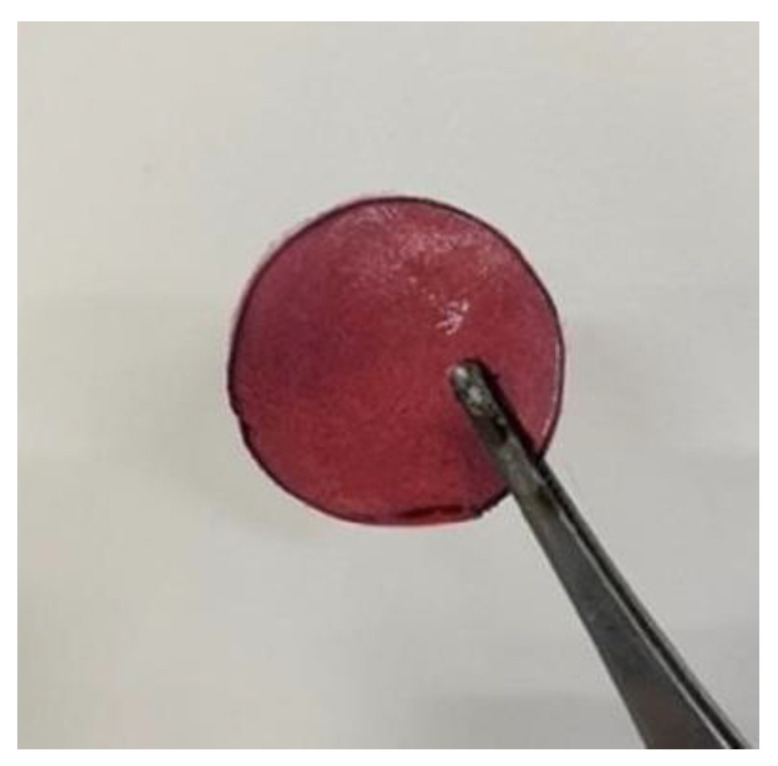
pH sensor.

**Figure 7:**
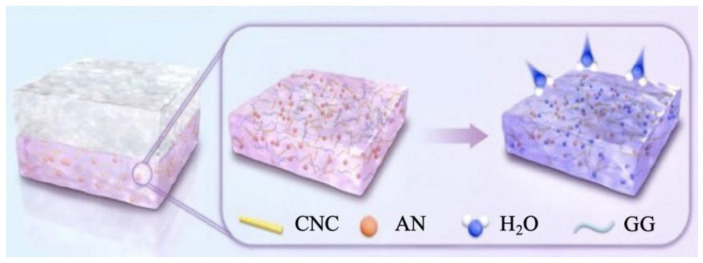
Schematic diagram of color change in pH sensor.

Finally, after the upper solution cooled, a robust bilayer hydrogel was formed (the samples labeled as TG/GG-CNC, TG/GG-CNC2, TG/GG-CNC4, TG/GG-CNC6, and TG/GG-CNC8, with CNC content of 0%, 2%, 4%, 6%, and 8%, respectively, as shown in Figure 6). The gel was dried into a film in a 50 °C vacuum drying oven, carefully peeled off, and then balanced for 24 h at 43% relative humidity (RH) for subsequent testing. Figure 7 illustrates the color-changing mechanism of the pH sensor. Table 2 summarizes the nomenclature and composition of the prepared composite membranes.

### 2.4. Structure Characterization of CNC

#### 2.4.1. Scanning Electron Microscopy (SEM)

The samples were fixed on the sample stage with double-sided tape, and then the powders of CNC and MCC were evenly spread on the tape surface, and the excess powder was removed. After the gold spraying, the samples were placed under the scanning electron microscope (SEM) to observe the microstructure.

#### 2.4.2. TEM Measurement

The 1–2 drops of CNC dispersion were added to the carbon support copper grid with a dropper and the excess liquid was removed by filter paper.

#### 2.4.3. XRD Spectrum Analysis

The crystallization characteristics of the samples were determined by D/max-2200 X-ray diffractometer (Cukα radiation, tube voltage 40 kV, tube current 30 mA, and scanning speed 5°/min), and the degree of crystallinity was calculated according to Segal method.

*CI* = (1 − *I_am_*/*I*_002_) × 100% (1)

where *CI* is the degree of crystallinity, *I_am_* is the intensity of the 2θ = 18° peak, and *I*_002_ is the intensity of the 2θ = 22.6° peak [18].

#### 2.4.4. FTIR Measurement

The absorption spectra of the samples were measured by Nicolet-iS50 Fourier transform infrared spectrometer (ATR mode) in the range of 500–4000 cm^−1^.

### 2.5. Performance Measurement of Intelligent Indicating Film

#### 2.5.1. Scanning Electron Microscopy (SEM) Observation

The thin film was fractured by liquid nitrogen, and the fracture surface was attached to the sample stage with conductive tape and gold spray. The fracture surface was observed and photographed by scanning electron microscope (SEM) with 10 kV accelerating voltage.

#### 2.5.2. FTIR Determination Fourier Transform Infrared (FTIR) Analysis

The infrared absorption spectra of the samples were measured by Nicolet-iS50 Fourier transform infrared spectrometer. The attenuation total reflection (ATR) mode was used, and the scanning range was set to 500–4000 cm^−1^ [19].

#### 2.5.3. UV–Vis Spectrum of the Thin Film in Different pH Buffer Solutions

The composite membrane was immersed in different pH buffer solutions for 30 min, and its UV–Vis absorption spectrum was measured immediately after removal, with the scanning wavelength range set from 300 to 600 nm.

#### 2.5.4. Determination of Water Contact Angle

The water contact angle of the smart indicator film was evaluated using the JC2000DM contact angle tester [20]. In the contact angle analysis, 10 μL of distilled water was dropped onto the surface of the film sample (2 × 2 cm) to detect its hydrophilicity/hydrophobicity.

#### 2.5.5. Water Vapor Transmission Test and Water Solubility (WS)

The determination of water vapor permeability (WVP) was performed in accordance with the ASTM E96-05 standard [21]. After cutting the film sample to be tested, it was sealed and fixed at the mouth of a centrifuge tube containing 20 mL of deionized water. The centrifuge tube was then placed in a constant temperature and humidity chamber for stabilization over a period of 7 days. During testing, the tube was removed every 12 h, weighed using a precision analytical balance, and the changes in its mass over time were systematically recorded. The water vapor permeability (WVP) was calculated using the following formula:

(2) $$WVP=\frac{△m\times x}{S\times △P\times t}$$

In the formula, Δm represents the mass change in the centrifuge tube over time t (g); x denotes the average thickness of the film (m); S indicates the effective transmittance area of the film (m^2^); ΔP is the vapor pressure difference across the film (Pa), with a value of 2339 Pa under pure water conditions at 20 °C; and t refers to the weighing interval (s). The final WVP value is calculated from the linear slope of the mass change during the steady-state diffusion phase.

Before conducting the water solubility (WS) measurement, the film was cut into 3 × 3 cm squares, and its mass was recorded as M1. The film sample was immersed in 50 mL of distilled water for 24 h, then dried at 105 °C until a constant weight was achieved. The weight after water removal was measured, and the mass was recorded as M2. The formula for calculating WS (Formula (3)) is as follows:

(3) $$WS(\%)=\frac{M1-M2}{M1}\;\times \;100$$

#### 2.5.6. Mechanical Properties

The film thickness was first measured using a handheld digital micrometer [22]. The tensile strength (TS) and elongation at break (EB) were determined with an XLW-PC intelligent electronic tensile testing machine. To evaluate the mechanical properties, the samples were initially stored in a desiccator containing saturated Mg(NO_3_)_2_ solution for two days. The film was then cut into 30 × 20 mm specimens, with the test parameters set to an initial gauge length of 20 mm and a tensile rate of 0.6 mm/s. The TS and EB values were calculated using Formulas (4) and (5), respectively.

(4) $$TS=\frac{F}{S}$$

(5) $$EB\left(\%\right)=100\%\times \frac{∆l}{l_{0}}$$

TS is the tensile strength (MPa), F is the maximum load (N), S is the initial cross-sectional area of the membrane sample, EB is the elongation at break (mm), ∆l is the elongation of the film (mm), and $l_{0}$ is the initial effective length of the film (mm) [23].

#### 2.5.7. Measurement of Light Stability

To evaluate the color stability of the films, three composite film samples (TG/GG, TG-AN/GG, and TG/TiO_2_-AN/GG) were exposed to UVA light (wavelength 320–400 nm, with a principal peak at λmax = 350 nm) for photodegradation. During the irradiation, surface images were captured every 2 h using an optical scanner. Subsequently, color parameters were extracted from the images using a custom program on the Matlab R2012a platform and quantitatively characterized by L (luminance), a (red-green value), b* (yellow-blue value), and total color difference (ΔE). The ΔE value was calculated according to the following Formula (6):

(6) $$∆E\;=\;\sqrt{{\left(L*-L_{0}\right)}^{2}+{\left(a*-a_{0}\right)}^{2}+{\left(b*-b_{0}\right)}^{2}}$$

where $L_{0}$, $a_{0}$, and $b_{0}$ are the initial color values of the film [24].

#### 2.5.8. Application of Slow-Release Fiber Membrane in Pork Preservation

This study employed a pH sensor with 6% aniline (AN) concentration for subsequent application validation. Fresh pork was cut into pieces and placed in a preservation box, with the pH sensor fixed on the inner wall. After sealing, a meat freshness comparison card was affixed to the outer surface of the packaging, and the entire package was stored at 4 °C. During the 5-day storage period, the pH value, volatile basic nitrogen (TVB-N), and total bacterial count of the pork samples were measured every 24 h according to GB 5009.237-2016, GB 5009.228-2016, and GB 4789.2-2022 [25,26,27], respectively. Simultaneously, the color changes in the sensor were recorded.

## 3. Results and Discussion

### 3.1. Establishment and Analysis of Response Surface Method Experimental Model

#### 3.1.1. Response Surface Design and Results

Table 3 shows the experimental design and results of response surface optimization.

#### 3.1.2. Establishment and Analysis of Yield Model

The software uses different models to deduce the input data and then finds the most suitable model for the data.

The results of comparing the fitting degree of the linear model, the interaction term model, and the quadratic term model show that the quadratic term model has the best fitting degree.

The results of the summary analysis of different models show that the quadratic model has better fitting degree, so the quadratic model is selected to further analyze the data.

To establish the quantitative relationship between the response variable (yield) and the experimental variables, the data in Table 3 were analyzed using Design-Expert 10.0.7 software. The response value (yield) was fitted with multiple regression using sulfuric acid concentration (A), temperature (B), and time (C) as independent variables, resulting in the following second-order polynomial equation:

Y = 62.42 − 12.27 × A + 11.24 × B + 10.93 × C − 0.69 × AB + 3.49 × AC − 8.79 × BC − 22.11 × A^2^ − 7.29 × B^2^ − 12.13 × C^2^

The response value of yield was used for multiple regression fitting. The regression model coefficients and significance test results are presented in Table 1, Table 2, Table 3, Table 4, Table 5, Table 6 and Table 7.

Table 8 and Table 9 provide further analysis of the model and its regression coefficients. As shown in Table 8, the regression model demonstrates statistical significance (*p* < 0.0001), while the residual term (*p* = 0.0955) is not significant (*p* > 0.05), indicating good model fit and the ability to predict corresponding regression values. The model’s R^2^ coefficient is 0.9892, with an adjusted R^2^ of 0.9752, suggesting that 97.52% of the data can be explained by the model. The coefficient of variation (CV) is 7.55, and the signal-to-noise ratio (SNR) is 24.552, indicating high reliability of the equation.

The F-value serves as a key metric for evaluating the impact of variables on response values. A higher F-value indicates greater contribution of the model components to the response. When the significance test probability *p* < 0.05, it demonstrates statistically significant influence of the variable on the response. Analysis of F-values and *p*-values reveals that the *p*-values for the first-order terms A, B, and C are all below 0.01, confirming that sulfuric acid concentration, temperature, and time have highly significant independent effects on yield, with their influence intensity ranked as sulfuric acid concentration (A) > temperature (B) > time (C). Among interaction effects, only the BC interaction (temperature × time) shows highly significant impact on yield (*p* <0.01), while the AB and AC interactions are not significant (*p* > 0.05).

**Figure 8:**
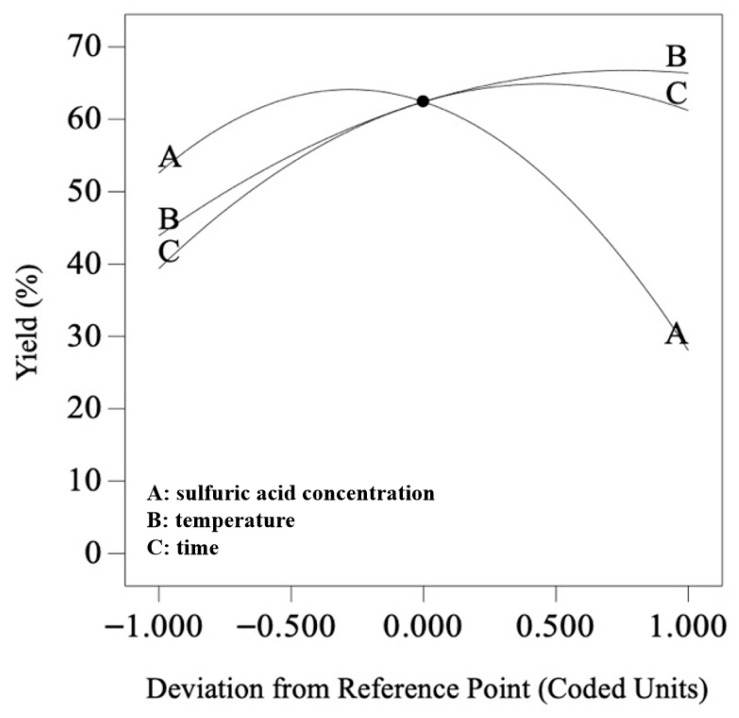
Single-factor trend chart of yield.

As shown in Figure 8, the yield increases first and then decreases with the increase in sulfuric acid concentration, the yield increases with the increase in temperature, and the tensile strength increases first and then flattens with the increase in time.

#### 3.1.3. Interaction of the Factors

Based on the regression equation and the shapes of the response surface plot and contour plots, the effects of sulfuric acid concentration, temperature, and time on the yield were analyzed. The response surface plot and contour plots effectively demonstrate the interactions among the three variables. By evaluating the steepness of the response surface plot, the degree of influence on the response value can be determined. A steeper response surface plot indicates a more pronounced interaction between the variables.

**Figure 9:**
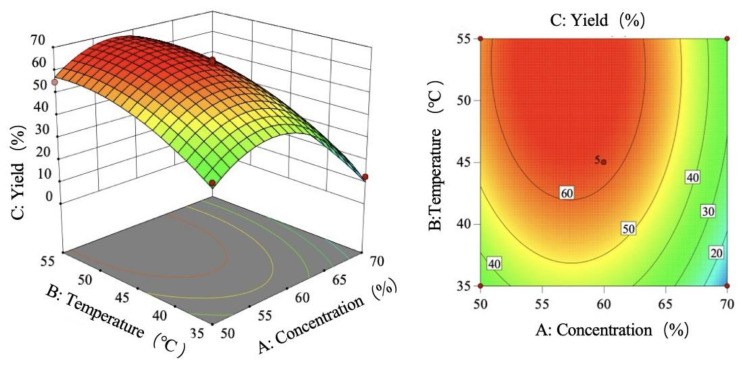
Response surface plot and contour plot of AB yield.

Figure 9 illustrates the interaction effect of sulfuric acid concentration (A) and temperature (B) on yield. The response surface reveals a monophasic pattern as follows: when temperature is fixed, the rate of yield change with concentration first increases and then decreases; when concentration is fixed, yield increases with temperature. The interaction analysis indicates that the yield peaks between 55 and 65% sulfuric acid concentration and 45–55 °C temperature. This graphical interpretation corroborates the interaction term significance findings in Table 8.

**Figure 10:**
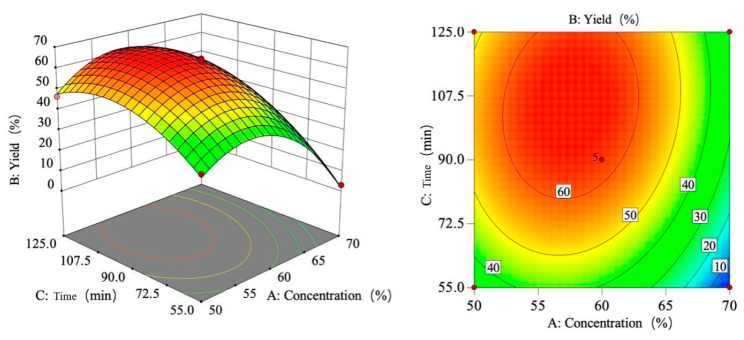
Response surface plot and contour plot of AC yield.

The interaction effect of sulfuric acid concentration and time (AC) on the yield is shown in Figure 10. Analysis of the response surface reveals that the rate of change in yield with sulfuric acid concentration first increases and then decreases, while the rate of change with time first increases and then levels off. This indicates that under the interaction of the two factors, the yield reaches its maximum when the sulfuric acid concentration is between 55 and 65% and the time is within 72.5–125 min. Comparing the slopes of the curves, it is found that the gradient change in the concentration direction is greater than that in the time direction, confirming that sulfuric acid concentration is a more critical influencing factor than time. The graphical analysis results are consistent with the conclusions of the variance analysis in Table 8.

**Figure 11:**
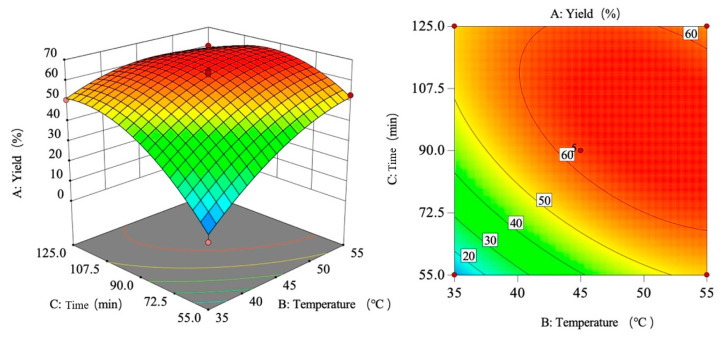
Response surface plot and contour plot of BC yield.

As shown in Figure 11, the interaction effect of temperature and time (BC) on the yield is analyzed. The response surface analysis reveals that when the temperature is lower, the yield increases first and then levels off as time progresses; when the temperature is higher, the yield initially rises and then declines with increasing time. Additionally, under short-term conditions, the yield increases with rising temperature; under long-term conditions, the yield also shows an initial increase followed by a decrease as temperature rises. This nonlinear relationship indicates a significant interaction effect between the two factors. The interaction analysis shows that the yield reaches its maximum within the range of 40–55 °C and 72.5–125 min. Furthermore, the slope of the surface along the temperature axis is steeper, indicating that the influence of temperature on the yield is greater than that of time. This graphical conclusion aligns with the results of the ANOVA in Table 7.

#### 3.1.4. Verification of Test Results

Based on the regression equation model, parameter optimization was conducted to maximize yield. The predicted optimal conditions were sulfuric acid concentration 57.25%, temperature 51.86 °C, and reaction time 95.65 min. Considering the precision of experimental operations, the conditions were adjusted to sulfuric acid concentration 57%, temperature 52 °C, and reaction time 96 min. Three parallel validation experiments were performed under these conditions, with results showing high agreement between experimental values and model predictions. The final determined optimal average yield was 65.86%.

### 3.2. Characterization Analysis of CNC Structure

#### 3.2.1. Observation by Scanning Electron Microscopy (SEM) and Transmission Electron Microscopy (TEM)

**Figure 12:**
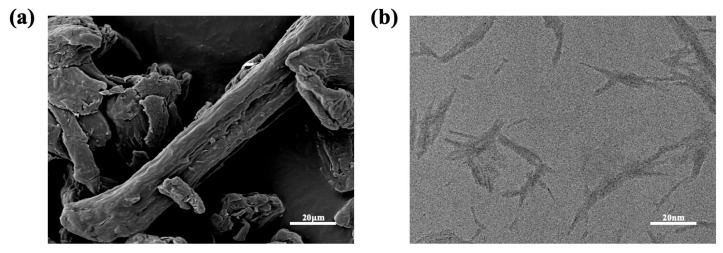
(**a**) SEM image of MCC, (**b**) TEM image of CNC.

As shown in Figure 12a, there is the scanning electron microscopy image of MCC and CNC, respectively. The images reveal that MCC appears as short rod-like structures with a diameter of approximately 20 μm. After acid hydrolysis and ultrasonic treatment, both the diameter and length of MCC were significantly reduced. According to Table 10, which compiles literature-reported CNC dimensions, the transmission electron microscopy results in Figure 12b indicate that the diameter of CNC is about 3 to 50 nm, with lengths ranging from 50 to 500 nm. Therefore, the CNC crystals prepared in this experiment are classified as CNC.

#### 3.2.2. XRD Spectrum Analysis

**Figure 13:**
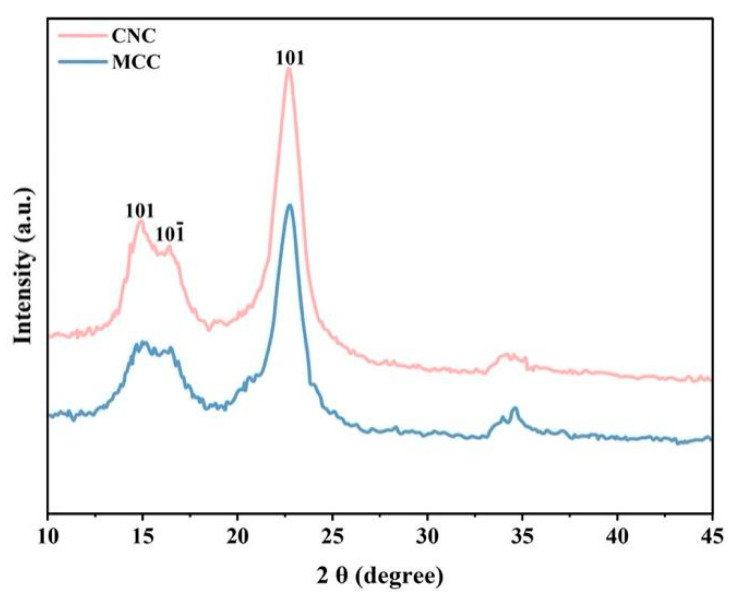
The X-ray diffraction patterns of MCC and CNC.

XRD analysis is a rapid analytical technique primarily used for the identification of crystalline materials and can be employed to evaluate the crystallinity of materials [28]. As shown in Figure 13, the X-ray diffraction patterns of MCC and CNC are displayed. From the figure, it can be seen that both MCC and CNC exhibit three diffraction peaks, which have similar characteristics and are located at 15.12°, 16.5°, and 22.75°, respectively. These are typical features of cellulose type I structure [29]. After acid hydrolysis treatment, the diffraction peak at 22.6° in CNC became sharper, with a significant increase in relative intensity. This indicates that hydrogen ions during hydrolysis penetrate the amorphous regions of cellulose, accelerating the cleavage of glycosidic bonds [30]. In this process, the amorphous cellulose is completely degraded, while the crystalline region, with lower accessibility and reactivity, only participates in surface reactions. The selective removal of amorphous components leads to a relative increase in the crystallinity of the remaining product (CNC).

The crystallinity of cellulose before and after hydrolysis was calculated using the Segal method [31].

#### 3.2.3. FTIR Measurement

**Figure 14:**
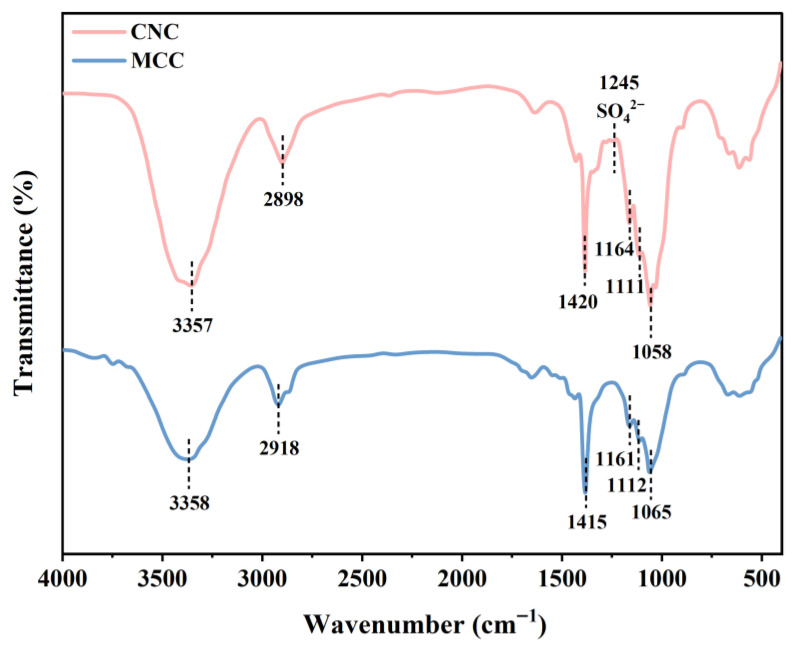
Infrared spectra of MCC and CNC.

ATR-FTIR spectroscopy was used to provide information about the presence or absence of specific functional groups, as well as the chemical structure of the biopolymer/polymer materials [32]. The FTIR spectra of MCC and CNC are shown in Figure 14. The characteristic absorption peaks of MCC correspond to the hydroxyl (-OH) stretching vibration at 3357 cm^−1^, the methylene (–CH_2_–) C–H stretching vibration at 2898 cm^−1^, and the cellulose alcohol C–O stretching vibration at 1058 cm^−1^. Near the main peak at 1058 cm^−1^, multiple weak shoulder peaks are observed, with the adjacent peaks at 1111 cm^−1^ and 1164 cm^−1^ corresponding to intramolecular C–O and C–C skeletal stretching vibrations, respectively. Additionally, a bending vibration peak of saturated C–H is detectable around 1420 cm^−1^ [33].

Compared to raw material microcrystalline cellulose (MCC), the sulfate hydrolysis method produces cellulose nanocellulose (sCNC) that retains its chemical backbone but exhibits surface structural changes. As shown in Figure 14, a new characteristic absorption peak appears at approximately 1220 cm^−1^, attributed to the asymmetric stretching vibration of sulfonic acid or sulfate groups (S=O). This clearly demonstrates that sulfate hydrolysis successfully introduces negatively charged sulfonic acid groups onto the CNC surface, indicating that the unique properties of CNC originate from nanoscale effects rather than chemical structural alterations [34]. Concurrently, the OH-stretching vibration peak of CNC becomes significantly narrower, revealing enhanced intramolecular hydrogen bonding. This phenomenon stems from the nanoscale size exposing abundant hydroxyl groups on the cellulose surface, thereby promoting stronger hydrogen bonding between these groups [35].

### 3.3. Performance Measurement of pH Sensor

#### 3.3.1. Scanning Electron Microscopy (SEM) Observation

**Figure 15:**
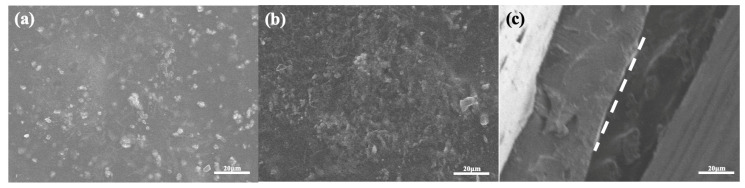
(**a**) SEM image of TG/TiO_2_ film, (**b**) SEM image of GG/AN/CNC film, and (**c**) cross-sectional SEM image of composite film.

The microstructure of the film can be observed by scanning electron microscopy (SEM) to evaluate its homogeneity, compactness, and component compatibility. As shown in Figure 15a, the TG layer with TiO_2_ added is relatively uneven, and wrinkles and some white protrusions can be seen on the surface. As shown in Figure 15b, the surface of the GG film with a small amount of CNC added is uniform, free of wrinkles, and relatively flat, indicating that GG molecules have good compatibility with an appropriate amount of CNC. Figure 15c shows the cross-sectional morphology of the TG/GG-CNC composite film, with a clear bilayer structure visible. This phase separation phenomenon between the TG layer and the GG layer is mainly attributed to the thermal irreversibility of the two hydrogels, which leads to their inability to mix during gelation and thermal drying processes. It is worth noting that no obvious gap was observed at the interface between the two layers, indicating that they may have undergone partial cross-linking through hydrogen bonds based on their polysaccharide characteristics. This kind of interface cross-linking is crucial for forming a stable bilayer film and avoids the peeling of a single film layer [36].

#### 3.3.2. FTIR Measurement

**Figure 16:**
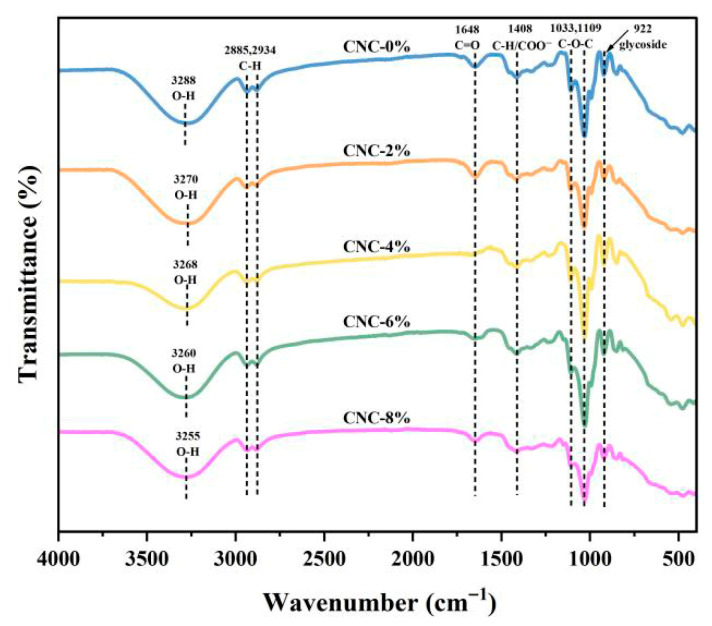
FTIR diagram of TG/GG composite film.

Due to the large specific surface area and surface activity of nanomaterials, they are usually added to compatible polymers to enhance the physical and chemical properties of the materials, such as mechanical strength and thermal stability g vibration of C–H. Figure 16 shows the FTIR of TG/GG. The peak at 1648 cm^−1^ is associated with the C=O stretching vibration, and the peaks at 1033 cm^−1^ and 1109 cm^−1^ correspond to the C–O–C stretching vibrations on the sugar ring. The 922 cm^−1^ peak indicates the stretching vibration of the pyran ring. After adding CNC and with increasing addition amounts (2%, 4%, 6%, and 8%), all samples exhibited vibration peaks similar to those in the TG/GG blank samples, with no significant changes in peak types or positions. This suggests that the interaction between CNC and TG/GG is purely physical without forming chemical bonds. Due to the high content of C–O–C groups in CNC, the intensity of the 1033 cm^−1^ and 1109 cm^−1^ peaks shows a marked increase compared to TG/GG. Simultaneously, as the CNC content increases, the O–H stretching vibration peaks gradually shift blueward to 3270 cm^−1^, 3268 cm^−1^, and 3255 cm^−1^, primarily due to the formation of strong hydrogen bonding interactions between CNC and TG/GG [37].

#### 3.3.3. Analysis of the Film in Different pH Buffer Solutions

**Figure 17:**
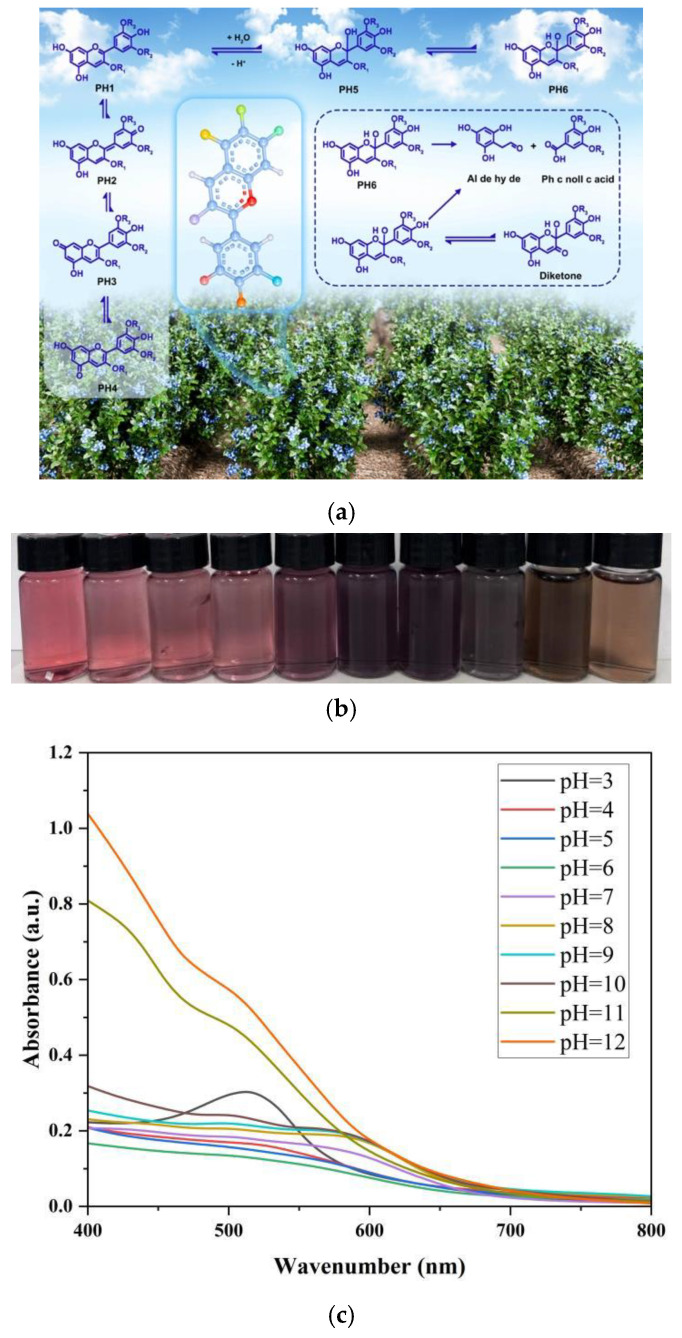
(**a**) Schematic diagram of the color change principle of blueberry anthocyanins. (**b**) Color changes in anthocyanins under different pH conditions. (**c**) Spectral changes in AN solution under different pH conditions.

Figure 17a illustrates the color-changing mechanism and structural changes in blueberry anthocyanins (AN) with pH variations. As shown in Figure 17b, the solution color transitions from red (pH 2–3) to pink (pH 4–5), light purple (pH 6–7), blue-purple (pH 8–11), and finally yellow (pH 12) as pH increases [38]. The corresponding UV–Vis spectrum (Figure 17c) reveals that when pH rises from 3 to 5, the maximum absorption peak at approximately 525 nm gradually weakens and shifts red-shifted to 570 nm. When pH increases from 6 to 10, this peak continues to shift blue-shifted to 580 nm with gradually enhanced intensity. Beyond pH 10, the absorption peak intensity drops sharply.

The color changes in the composite membrane prepared in this study in different pH buffer solutions (Table 11) were consistent with the behavior of the AN solution, confirming the application potential of this composite membrane in meat freshness monitoring.

#### 3.3.4. Determination of Water Contact Angle

**Figure 18:**
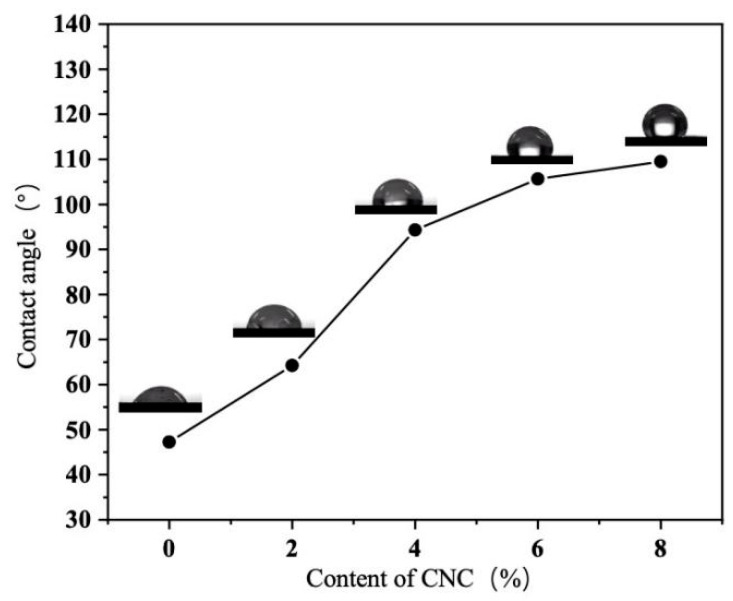
Image of water contact angle test of composite film.

Figure 18 illustrates the surface wettability of the composite film. When 4% CNC was added, the contact angle increased significantly from 47.2° in the blank sample to 94.2°, indicating a transition from hydrophilic to hydrophobic surface properties. Subsequent increases in CNC content resulted in a gradual flattening of the contact angle growth. According to Young’s equation, a contact angle exceeding 90° is classified as non-wetting (hydrophobic). This effect aligns with previous studies on CNC in other polysaccharide-based composite membranes, such as agar [39], starch [40], and sodium alginate [41]. This study demonstrates that CNC, as a high-performance bio-nanofiller, exhibits a unique dual mechanism for regulating the wettability of bio-based films, providing both hydrophobic components and rough surface structures [42]. The formation of such hydrophobic surfaces holds significant implications for the meat freshness monitoring film in this research. It may help reduce direct interference from water vapor on the sensing unit and, to some extent, delay the migration or loss of water-soluble components (e.g., anthocyanins), thereby potentially enhancing the sensor’s stability.

#### 3.3.5. Analysis of Water Vapor Transmission Rate

**Figure 19:**
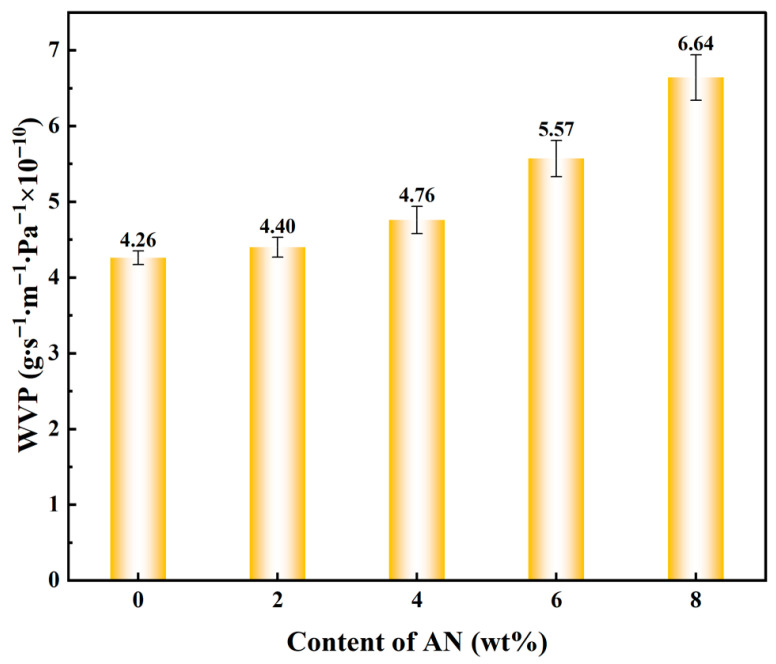
Test images of water vapor transmission rate with different anthocyanin contents.

Water vapor permeability (WVP) is a critical parameter for evaluating the moisture barrier performance of hydrogels, which holds significant implications for their application in food packaging. The relationship between anthocyanin content and WVP is shown in Figure 19. Studies indicate that the addition of anthocyanins significantly affects the WVP of TG/GG-based hydrogels, with a positive correlation observed between the two. Specifically, as anthocyanin content increases, the material’s moisture resistance gradually decreases. The anthocyanin-free TG/GG substrate inherently exhibits low WVP, demonstrating excellent moisture barrier properties. When a small amount of AN is added, the increase in WVP is limited, suggesting no significant changes in the membrane structure at this stage. However, as anthocyanin content continues to rise, WVP shows a sustained increase, reaching a peak value at 8% anthocyanin concentration.

The primary mechanism by which anthocyanins elevate water vapor permeability (WVP) stems from their alteration of the hydrogel’s microstructure. As natural polyphenolic compounds, anthocyanins not only modify the hydrogel’s microstructure but also increase the porosity of the gel network. When anthocyanins aggregate to form micron-scale phase separation or granular features, they create additional channels within the gel, thereby shortening water vapor pathways and facilitating moisture migration.

Therefore, while utilizing anthocyanins to endow hydrogels with functional properties, attention must be paid to their adverse effects on moisture barrier performance. Subsequent approaches, such as improving anthocyanin dispersibility, optimizing cross-linked networks, or adopting composite structure designs, can balance the functionality and barrier properties of the materials, thereby expanding their application potential in the field of active packaging.

**Figure 20:**
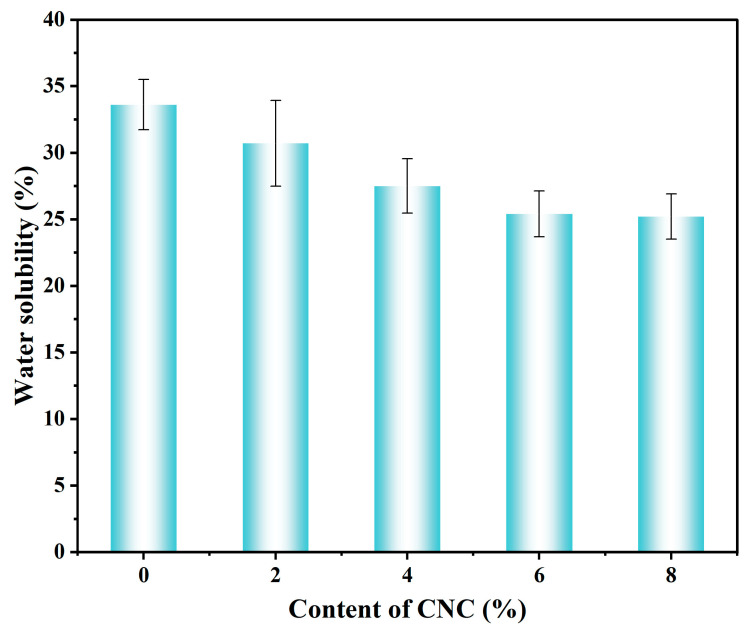
Test images of water solubility rate with different CNC contents.

The water solubility rate of different CNC contents is shown in Figure 20. Under the same measurement conditions, as the content of CNC in the lower layer increases, the water solubility (WS) of the double-layer film shows a significant downward trend. The WS decreases from 33.6% (0% CNC) to 25.4% (6% CNC). This trend indicates that through hydrogen bond interactions, CNC forms a denser nanoscale network with the TG/GG matrix, reducing the entry and swelling of water molecules, thereby lowering the dissolution amount. Additionally, the dense structure of the upper layer containing TiO_2_ further hinders water penetration, resulting in a WS of the double-layer system being lower than that of the single-layer hydrophilic film. However, when the CNC content reaches 8% (WS 25.2%), due to the aggregation and other phenomena in the double-layer film, the water solubility slows down.

#### 3.3.6. Mechanical Properties Measurement

**Figure 21:**
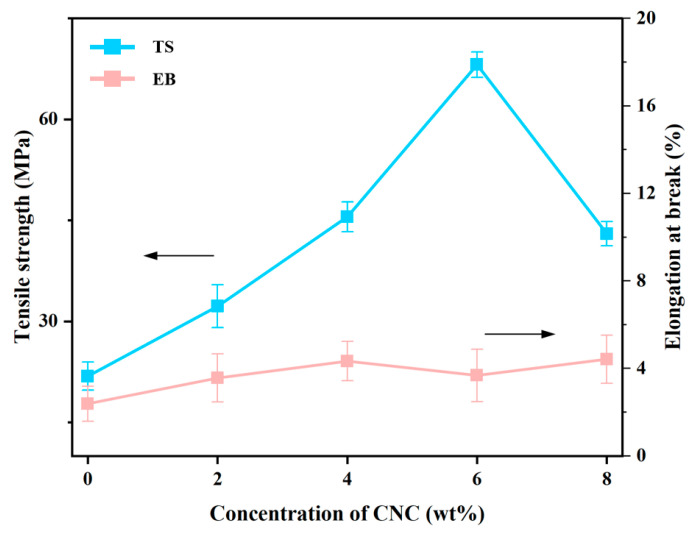
Mechanical properties of composite membrane materials.

Figure 21 displays the tensile strength and elongation at break of the composite film. The TG/GG blank samples exhibited a typical non-monotonic variation pattern after CNC addition. When the CNC content increased from 0% to 6%, the tensile strength rose from 22 MPa to 72 MPa, while the elongation at break surged from 3.75% to 4.17%, demonstrating significant simultaneous improvement in both strength and toughness. This synergistic effect primarily stems from the physical network formed by uniform CNC dispersion, which effectively bears and transmits stress [43]. Additionally, the abundant hydroxyl groups on the CNC surface form dense hydrogen bond networks with polar groups on the TG/GG molecular chains. These strong interfacial interactions not only serve as physical crosslinking points to enhance stiffness but also promote chain reorganization and orientation during tensile testing, thereby significantly improving ductility alongside tensile strength. However, when the CNC content reached 8%, an inflection point emerged (tensile strength: 42 MPa, elongation: 4.7%), likely due to agglomeration of CNC at high concentrations. This aggregation easily causes stress concentration, reducing stress transfer efficiency [44,45]. This phenomenon is consistent with the functional response of alginate-gelatin-nanocrystalline cellulose injectable hydrogels to the delivery of cells and bioactive molecules/CNC, carrageenan/CNC [46]. Therefore, the optimal CNC content is about 6%.

#### 3.3.7. Analysis of Light Stability

As a freshness indicator label, the color stability of the film is critical [47]. The UV (100–400 nm) band of the solar spectrum comprises the following three ranges: UVA (400–315 nm), UVB (315–280 nm), and UVC (280–100 nm). Since nearly all UVC-range light and most UVB-range light are absorbed by ozone in the Earth’s stratosphere [48], UVA (315–400 nm) dominates in real-world conditions. Therefore, this study focuses on the effects of UVA (λ = 320–400 nm, λmax = 350 nm).

**Figure 22:**
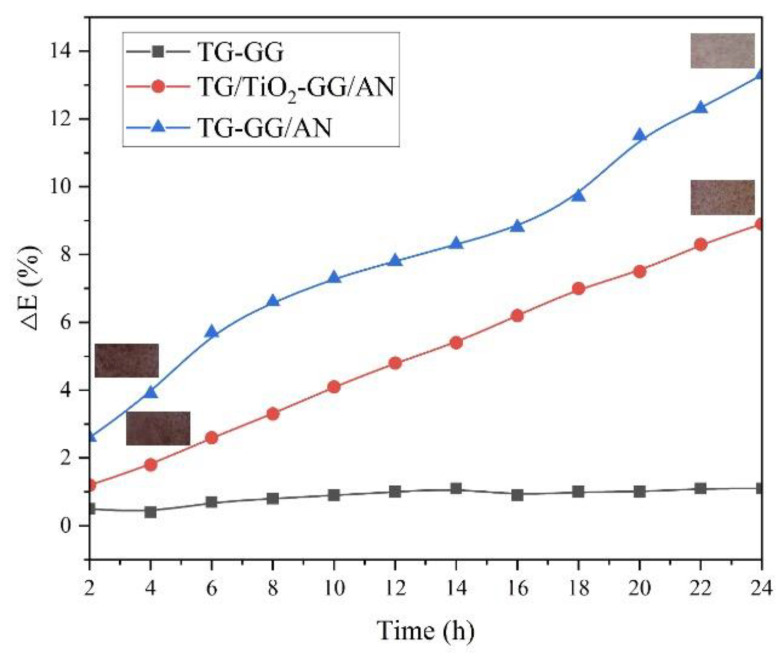
Image of the photo-stability test of composite film.

After 24 h of UVA exposure, the film’s color change is shown in Figure 22. The TiO_2_-free film, due to anthocyanin (AN) photodegradation, shifted from light purple to pink–purple. Quantitative color difference analysis revealed a ΔE value of 13.3%. In contrast, the TiO_2_-added film exhibited a ΔE value of only 8.9%, demonstrating significantly improved stability (note: ΔE values typically below 5 are barely perceptible to the human eye [49]).

This enhancement is primarily attributed to the dual protective mechanism of rutile-type TiO_2_ nanoparticles. As wide-bandgap semiconductors, they can absorb ultraviolet (UV) energy through intrinsic absorption [50]. Moreover, nanoparticles further attenuate UV radiation via scattering effects. Additionally, compared to anatase-type materials, rutile-type materials exhibit significantly lower photocatalytic activity, which substantially reduces polymer degradation caused by reactive oxygen species (ROS) generation and minimizes potential food safety risks, making them more suitable for food packaging applications.

#### 3.3.8. Visual Intelligence Perception of Pork Freshness

**Figure 23:**
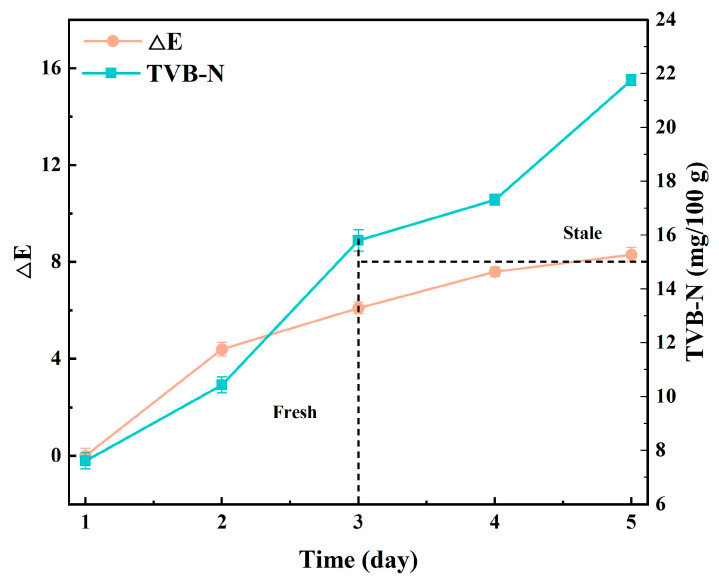
The graph of the changes in TVB-N value and color difference value ΔE of intelligent composite film of pork with different storage days.

This study applied anthocyanin-containing hydrogels as smart sensing membranes for pork freshness monitoring, demonstrating excellent visual and sensing performance. The trend of TVB-N value and color difference value of pork during storage is shown in Figure 23. Experimental data revealed that the TVB-N value of fresh pork at the initial storage stage was 7.61 mg/100 g. With prolonged storage, by 48 h, the TVB-N increased to 10.43 mg/100 g at a pH of 5.8, with a color difference (ΔE) of 4.4, indicating the meat sample still met food safety standards (GB 2707-2016) [51]. By 96 h, the TVB-N further rose to 17.31 mg/100 g (exceeding the edible limit), with the pH rising to 6.5 and ΔE reaching 7.6, accompanied by a reddish–purple color change, indicating a significant decline in freshness and reaching the unpalatable standard. After 120 h of storage, the TVB-N reached 21.76 mg/100 g, with the pork becoming spoiled and emitting an unpleasant odor. At this stage, the pH was 7.2, ΔE increased significantly to 8.3, and the color turned light purple, confirming marked spoilage with an unpleasant odor.

The study demonstrates that anthocyanins integrated with membrane materials can effectively indicate pH changes caused by microbial metabolism through color variations, maintaining synchronization with TVB-N value trends. As pork quality declines due to rising pH levels during storage, the membrane’s color response accurately reflects spoilage progression, providing an eco-friendly and intuitive solution for food freshness monitoring. This research establishes theoretical foundations and practical references for developing natural pigment-based intelligent sensing technologies and food quality monitoring methods, significantly advancing food safety management innovations.

## 4. Conclusions

This study developed a dual-layer structural indicator membrane, with the base layer consisting of a composite film of junction cold adhesive/anthocyanin/nanocellulose serving as the sensing unit, and titanium dioxide as the UV isolation layer. The system is designed for real-time, non-destructive freshness monitoring of meat products. The UV isolation layer effectively addresses anthocyanin instability under light exposure while extending the composite film’s service life in real-world storage environments. Through response surface methodology, the preparation process of CNC was optimized to achieve efficient extraction of nanocellulose and its incorporation into the sensing layer, significantly improving mechanical properties, hydrophobicity, and thermal stability compared to conventional anthocyanin polymer composite films. Results showed that as CNC content increased, dispersion gradually decreased. When added at 8%, the tensile strength of the membrane material dropped to 42 MPa. The impact of CNC content on elongation at break and thermal stability was minimal, with the material demonstrating optimal comprehensive performance at 6% CNC content.

The dual-layer membrane exhibits a sensitive color response to pH fluctuations caused by microbial proliferation, enabling direct monitoring of food freshness. This indicator label offers advantages including low manufacturing costs, environmental friendliness, and ease of monitoring. It is suitable for quality control in both prepackaged and conventional cold-chain food supply chains, while also providing consumers and regulators with an effective food safety monitoring tool. This study presents an intelligent packaging solution with practical application potential for addressing freshness control challenges in modern food industry, further strengthening consumers’ food safety defenses.

It should be noted that agglomeration of TiO_2_ nanoparticles was observed on the surface of the TG/TiO_2_ UV isolation layer in this study. This phenomenon may lead to uneven UV shielding distribution at the microscopic level and compromise the mechanical integrity of the film surface. Although the current design has achieved basic functionality, future efforts should focus on optimizing dispersion processes (e.g., ultrasonic treatment or surface modification) to enhance TiO_2_ dispersion uniformity, which will be key to improving the film’s long-term performance.

## Figures and Tables

**Table 1:** Experimental design for response surface analysis with three factors and three levels.

Factor	Horizontal		
	−1	0	1
A—sulfuric acid concentration (%)	50	60	70
B—temperature (℃)	35	45	55
C—time (min)	55	90	125

**Table 2:** The prepared composite membrane materials are abbreviated as and their components.

Nomination of Composite Films	Composite Membrane Components
TG/GG-CNC	Tara glue (TiO_2_ layer)/junction cold glue (anthocyanin layer)—no cellulose added
TG/GG-CNC2	Tara glue (TiO_2_ layer)/junction cold glue (anthocyanin layer)—added 2% cellulose
TG/GG-CNC4	Tara glue (TiO_2_ layer)/junction cold glue (anthocyanin layer)—added 4% cellulose
TG/GG-CNC6	Tara glue (TiO_2_ layer)/junction cold glue (anthocyanin layer)—added 6% cellulose
TG/GG-CNC8	Tara glue (TiO_2_ layer)/junction cold glue (anthocyanin layer)—added 8% cellulose
TG/GG	Tara glue–set cold glue blank sample
TG-221AN/GG	Tara gum–anthocyanin/gelatin
TG/TiO_2_-AN/GG	Tara gum/TiO_2_–anthocyanin/vermicell

**Table 3:** Experimental design and results of response surface optimization.

Test Number	A—Sulfuric Acid Concentration	B—Temperature	C—Time	Yield (%)	Predicted Accuracy (%)
1	50	35	90	35.56	33.37
2	70	35	90	12.5	10.21
3	50	55	90	54.94	57.23
4	70	55	90	29.11	31.3
5	50	45	55	34.47	33.02
6	70	45	55	2.85	1.49
7	50	45	125	46.53	47.89
8	70	45	125	28.89	30.34
9	60	35	55	8.39	12.04
10	60	55	55	52.93	52.1
11	60	35	125	50.66	51.49
12	60	55	125	60.03	56.38
13	60	45	90	61.15	62.42
14	60	45	90	64.58	62.42
15	60	45	90	59.5	62.42
16	60	45	90	63.02	62.42
17	60	45	90	63.87	62.42

**Table 4:** Comprehensive analysis of fit degree.

Type	Continuous *p*-Value	Misfit *p* Value	Regulation of R2	Predict R2	Bear Fruit
Linear	0.0366	0.0003	0.3460	0.1623	-
2FI	0.7758	0.0002	0.2349	−0.3150	
Quadratic	<0.0001	0.0955	0.9752	0.8635	Suggested

**Table 5:** Variance analysis of multiple models.

Variance Source	Quadratic Sum	Free Degree	Mean Square	F Price	Probability > F	Bear Fruit
Mean vs. Total	31,259.52	1	31,259.52			
Linear vs. Mean	3170.80	3	1056.93	3.82	0.0366	
2FI vs. Linear	360.01	3	120.00	0.37	0.7758	
Quadratic vs. 2FI	3161.96	3	1053.99	100.65	<0.0001	Suggested
Cubic vs. Quadratic	56.03	3	18.68	4.33	0.0955	
Residual	17.27	4	4.32			
Amount to	38,025.60	17	2236.80			

**Table 6:** Out-of-fit validation table.

Variance Source	Quadratic Sum	Free Degree	Mean Square	F Price	Probability > F	Bear Fruit
Linear	3578.00	9	397.56	92.09	0.0003	
2FI	3217.99	6	536.33	124.24	0.0002	
Quadratic	56.03	3	18.68	4.33	0.0955	Suggested
Pure Error	17.27	4	4.32			

**Table 7:** Summary statistics of the model.

Type	Sample Standard Deviation	Degree of Fitting	Corrected Fit	Predicted Goodness of Fit	Definition	Bear Fruit
Linear	16.63	0.4686	0.3460	0.1623	5667.64	
2FI	17.99	0.5218	0.2349	−0.3150	8897.45	
Quadratic	3.24	0.9892	0.9752	0.8635	923.53	Suggested
Cubic	2.08	0.9974	0.9898		+	

**Table 8:** Regression analysis results of yield model and regression coefficients.

Source	Sum of Squares of Deviations	Free Degree	Mean Square	F Price	*p* Price	Conspicuousness
Model	6692.77	9	743.64	71.01	<0.0001	**
A—sulfuric acid concentration	1204.18	1	1204.18	114.99	<0.0001	**
B—temperature	1010.25	1	1010.25	96.47	<0.0001	**
C—time	956.38	1	956.38	91.33	<0.0001	**
AB	1.92	1	1.92	0.18	0.6815	
AC	48.86	1	48.86	4.67	0.0676	
BC	309.23	1	309.23	29.53	0.0010	**
A^2^	2057.77	1	2057.77	196.51	<0.0001	**
B^2^	223.73	1	223.73	21.37	0.0024	**
C^2^	619.73	1	619.73	59.18	0.0001	**
Residual	73.30	7	10.47			
Fictitious term	56.03	3	18.68	4.33	0.0955	ns
Pure error	17.27	4	4.32			
Sum	6766.08	16				

Note: *p* < 0.01 indicates extremely significant (marked with **), *p* < 0.05 indicates significant (marked with *), and *p* > 0.05 indicates non-significant (marked with ns).

**Table 9:** Correlation analysis results of the model.

Project	Numeric Value	Project	Numeric Value
Standard error	3.24	Model correlation coefficient (R2)	0.9892
Mean	42.88	Adjusted coefficient of determination (R2Adj)	0.9752
Coefficient of variation/%	7.55	Predetermined coefficient of determination (R2Pre)	0.8635
Sum of squared prediction errors	923.53	Relative accuracy	24.552

**Table 10:** Summary of CNC types and parameters.

Granular Type	Surface	Microscopic Morphology	Size	Crystallinity
MCC	White opaque powder	Dense irregular granule	10–50 μm	55–75%
MFC/NFC	Opaque to translucent gel or paste	Long and entangled fiber meshes	10–100 nm	50–70%
NFC	Opaque to translucent gel or paste	Long and entangled fiber meshes	5–60 nm	-
CNC	Suspension with milky light, drying to form a transparent film	Short rod-like rigid nanocrystals	3–50 nm, length 50–500 nm	54–88%
t-CNC	Suspension or dispersion in an organic solvent	Short stick, surface chemical group changed	3–50 nm	80–90%

**Table 11:** Color changes in composite membrane in different pH buffer solutions.

pH	Simple	*L*	*a*	*b*	pH	Simple	*L*	*a*	*b*
**pH3**	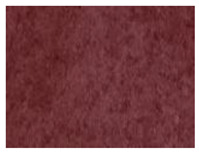	49.5	38.8	4.5	**pH4**	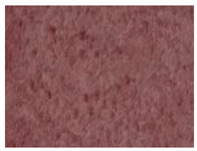	53.6	31.2	5.2
**pH5**	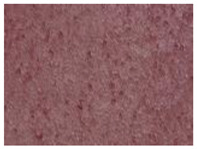	48.2	21.1	5.3	**pH6**	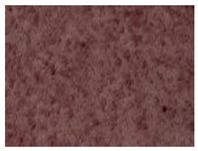	45.9	14.7	1.9
**pH7**	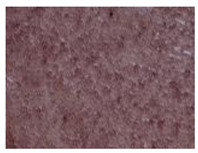	40.2	19.3	4.1	**pH8**	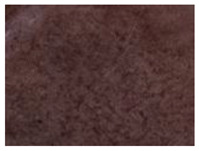	40.1	16.8	−4.3
**pH9**	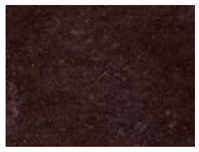	29.2	2.1	−3.2	**pH10**	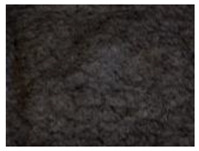	43.4	−0.3	−5.3
**pH11**	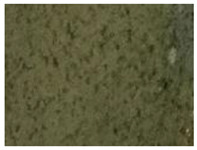	53.1	−0.5	7.2	**pH12**	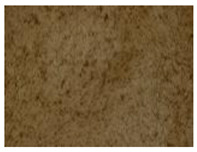	55.2	0.4	9.1

## Data Availability

The original contributions presented in this study are included in the article. Further inquiries can be directed to the corresponding authors.
